# Direct Oxidation of Glucose to Glucaric Acid Using Bimetallic AuPt/ZrO_2_ Nanocatalysts

**DOI:** 10.1021/acsanm.5c03743

**Published:** 2025-10-23

**Authors:** Joanna Elzbieta Olszowka, Abdul Selim, Žan Lavrič, Janvit Teržan, Ana Kroflič, Miha Grilc, Blaž Likozar, Jaroslav Kupčík, Esther de Prado, Jan Plsek, Eliska Mikyskova, Jaroslava Moravkova, Matej Huš, Stefan Vajda

**Affiliations:** † Department of Nanocatalysis, J. Heyrovský Institute of Physical Chemistry of the CAS, Dolejškova 2155/3, 182 23 Prague, Czech Republic; ‡ Department of Catalysis and Chemical Reaction Engineering, 68913 National Institute of Chemistry, Hajdrihova 19, 1000 Ljubljana, Slovenia; § University of Nova Gorica, Vipavska 13, SI-5000 Nova Gorica, Slovenia; ∥ Department of Material Analysis, 86889 Institute of Physics of the CAS, Na Slovance 1999/2, 182 00 Prague, Czech Republic; ⊥ Centre of Instrumental Techniques (CIT), Institute of Inorganic Chemistry of the Czech Academy of Sciences, Husinec-Řež 1001, 250 68 Husinec-Řež, Czech Republic; # Department of Low-Dimensional Systems, J. Heyrovský Institute of Physical Chemistry of the CAS, Dolejškova 2155/3, 182 23 Prague, Czech Republic; ∇ Center for Innovations in the Field of Nanomaterials and Nanotechnologies, J. Heyrovský Institute of Physical Chemistry of the CAS, Dolejškova 2155/3, 182 23 Prague, Czech Republic; ○ Department of Structure and Dynamics in Catalysis, J. Heyrovský Institute of Physical Chemistry of the CAS, Dolejškova 2155/3, 182 23 Prague, Czech Republic; ◆ Association for Technical Culture of Slovenia (ZOTKS), Zaloška 65, SI-1000 Ljubljana, Slovenia; ¶ Institute for the Protection of Cultural Heritage of Slovenia (ZVKDS), Poljanska 40, SI-1000 Ljubljana, Slovenia

**Keywords:** AuPt nanocatalyst, nanoalloy, bimetallics, biomass conversion, glucose to glucaric acid

## Abstract

Transforming biobased resources, such as glucose, into value-added chemicals is a crucial step in utilizing biomass. Herein, we report on the one-pot conversion of glucose to glucaric acid, by selectively steering the oxidation of glucose from the typical production of gluconic acid toward the production of glucaric acid, using monometallic Au and Pt and bimetallic AuPt nanocatalysts supported on zirconia. While the monometallic catalysts promote the production of gluconic acid, bimetallic catalysts favor the direct formation of glucaric acid from glucose, with efficacy depending on the Au/Pt ratio, reaching up to 44% selectivity with the Au_67%_Pt_33%_@ZrO_2_ catalyst. Theoretical calculations confirm the formation of alloys, as experimentally evidenced by EDX-mapping and HR-TEM imaging.

## Graphical abstract

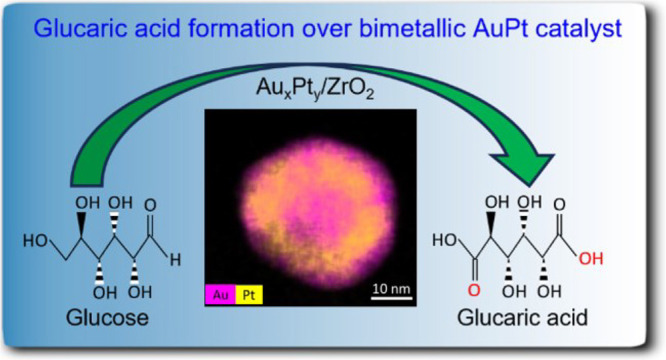

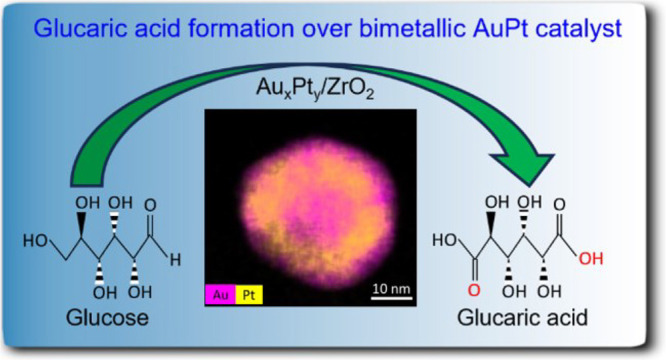

## 1 Introduction

The conversion of renewable natural resources presents an appealing and sustainable approach to reducing fossil fuel consumption, especially the conversion of waste biomass into energy, which is poised to significantly contribute to effective waste management practices and reduce their environmental impact.^1^ Extensive research efforts in academia and industry have focused on the catalytic conversion of carbohydrate-based biomass, with particular emphasis on glucose (Glc), the most abundant natural feedstock, for the production of renewable polymers, lactic acid, or aromatic monomers.^2,3^ Both gluconic acid (GO) and glucaric acid (GA) are key intermediates in biomass-to-chemicals conversion processes.^3–7^ GO and its salts are important compounds with various applications in the food and beverage, pharmaceutical, textile, and papermaking industries.^8^ GA and its salts are used as components in liquid detergents,^9^ as substitutes for phosphates,^9^ food additives,^5^ biobased adipic acid, hyperbranched polyester,^10,11^ pharmaceutical and therapeutic applications.^12^

GA is typically produced industrially through the oxidation of Glc using nitric acid, in a complex and environmentally problematic process.^13^ Current technologies suffer from several drawbacks, including the use of hazardous reagents, challenges in separating and recycling homogeneous catalysts, and the generation of toxic coproducts.^13^ Another drawback of using nitric acid is that it produces N_2_O, which has a 180 times greater global warming potential than CO_2_.^14^ Hence, developing more sustainable and efficient routes is important for large-scale applications. Several approaches have been proposed for the direct catalytic selective oxidation of Glc, including fermentation,^4^ electrocatalytic^15^ and photocatalytic oxidation,^16^ oxidation using air/oxygen,^6,17,18^ or related nitroxyl radical oxidants,^19^ which could lead to the development of more sustainable methods with a lower environmental impact.

In addition to GO and GA, several undesired products, such as tartaric acid (TA), tartronic acid (TO), glycolic acid (GLY), oxalic acid (OX), and formic acid (FA), were found by ion chromatography during the oxidation of Glc. A tentative pathway for their formation is shown in Scheme 1.^20^

**Scheme 1:**
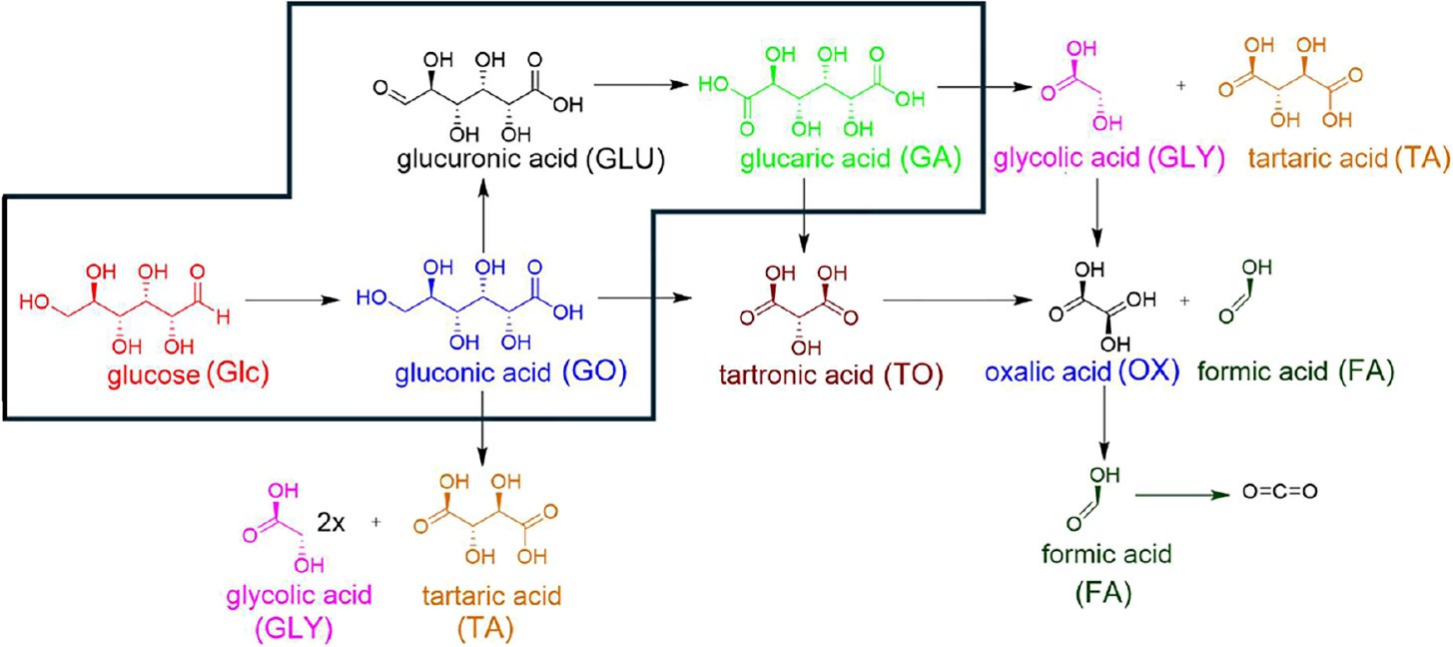
Possible Pathways for Glucose Oxidation with the Desired Route Shown in a Box (Glc → GO → GLU → GA)

The production of GA from Glc involves two key oxidation steps: in the first step, Glc undergoes oxidation of its aldehyde group through either C–H bond activation or C=O hydration into a geminal alcohol intermediate. In the subsequent step, GO is further oxidized to GA via GLU as an intermediate. Oxidation of GO to GLU is dependent on the C–H bond cleavage in the −CH_2_OH group, while the O–H bond cleavage proceeds readily.^20^ Oxidation of GLU to GA entails an aldehyde group oxidation, similar to the transformation of Glc to GO.

Lumping these steps into experimentally observable intermediates, this transformation involves activation of the C=O bond and oxidation of hydroxyl groups to carboxyl groups. Therefore, maintaining a high selectivity for GO is crucial for efficiently producing GA.

In this context, the development of new catalysts that enable one-pot Glc oxidation to GA under base-free and mild oxidation conditions has gained much interest.^17^ Gold catalysts were reported to be selective toward GO rather than GA in the absence of alkali additives.^21^ Megías-Sayago et al. studied Au-based catalysts for Glc conversion, reaching around 80% with a selectivity to GO of ∼100% at 40 °C under base-free conditions.^22^ Cao et al. demonstrated that the Au/TiO_2_ catalyst had the highest activity in Glc conversion (71%) and GO yield (67%) after 1 h at under 160 °C and 3 bar_g_ O_2_ conditions.^21^ Repo and co-workers reported Glc oxidation to GA over an Au/Al_2_O_3_ catalyst in the presence of base H_2_O_2_ as an oxidant, resulting in a 76% yield of GA under microwave irradiation at 120 °C for 10 min.^23^ On the other hand, Pt catalysts supported on activated carbon, SiO_2_, and ZrO_2_ provide more than 60% yield of GA during Glc oxidation under base-free conditions.^24^

There has been considerable research on Au/Pt catalysts for the liquid phase oxidation of a variety of polyols, such as sorbitol^25^ and glycerol,^26^ because they are more stable and give higher specific selectivity than their monometallic counterparts. The method of preparing these catalysts is well-known to affect the size and interaction of metal particles, as well as the structure and homogeneity of a bimetallic system (e.g., nanoparticles segregated into cores-shells, multilayered nanoparticles, and/or mixed nanoalloys), along with their properties.^27^ Jin et al. studied the influence of the bimetallic catalysts of PtPd and Pt–Cu supported on TiO_2_ to oxidize Glc with O_2_ under a basic medium. The results showed that PtPd-TiO_2_ catalysts exhibited significantly higher catalytic activity and improved selectivity to GA (44%) in the oxidation of Glc solution in water compared to their monometallic counterparts.^28^ Nevertheless, in the presence of a base, it promotes unfavorable side reactions leading to C–C and C–O cleavage, which adversely affects GA selectivity.^12^ Shi et al. reported that Pt–Cu/TiO_2_ bimetallic catalyst showed 92% conversion and ∼60% GA selectivity in one-step Glc oxidation under base-free conditions.^29^ Additionally, Elie et al. reported that AuPt/ZrO_2_ bimetallic catalyst gives a 50% yield of GA at 100 °C under 40 bar_g_ O_2_ without adding alkali.^30^ Recently, Potrzebowska et al. reported that ZrO_2_-supported AuPt catalysts are stable in the oxidation of Glc to GA under base-free conditions.^31^ They showed how the preparation process influences the AuPt alloy formation (Au/Pt ratio 1.11) and demonstrated that ultrasonication during preparation increases the conversion of Glc to GA. The yield was 71% with fewer side products at 100 °C under 40 bar_g_ O_2_. Au:Pt catalysts with different ratios were also studied on TiO_2_ support for glucose overoxidation to TA.^31^ Similar to the findings of work,^32^ alloy formation was identified as crucial for high catalytic performance; however, in both cases, the presence of the alloy was inferred only indirectly (via UV–Vis studies and Vegard’s law calculations).

Motivated by our initial exploratory combined experimental and theoretical studies, which indicated the potential of using small-sized bimetallic AuPt nanoparticles for the production of GA from Glc,^20^ the current study is focused on controlling catalyst performance by establishing the Au-to-Pt ratio to increase selectivity in the one-pot production of GA from Glc. The structure–function relationship in these catalysts, including the role of individual metal components and their synergy, is addressed in combination with catalyst testing, complemented by the characterization of the catalysts and theoretical calculations.

## 2 Experimental Section

Chemicals used for the synthesis, such as gold­(III) chloride trihydrate [HAuCl_4_·3H_2_O, 99.9%], chloroplatinic acid hydrate [H_2_PtCl_6_·*x*H_2_O, 99.9%], hydrazine hydrate (NH_2_–NH_2_·H_2_O, 50–60% aqueous solution), NH_4_OH solution, and glucose, were purchased from Sigma-Aldrich. All chemicals were of analytical grade and were used without further pretreatment. Deionized water was used as a solvent during the experiments. The preparation of monometallic and bimetallic Au, Pt nanocatalysts supported on ZrO_2_ was done by using hydrazine hydrate as a reducing agent. One g of the support (ZrO_2_) was dispersed in 20 mL of H_2_O, and separately variable amounts of Au and Pt salts corresponding to the desired loadings were dissolved in 5 mL of H_2_O (see Table S1). Next, the solution and dispersion were mixed under vigorous stirring. After 10 min, 1.5 mL of a 2.5% NH_4_OH aqueous solution was added, and the suspension was stirred for another 2 h at room temperature. Then 0.256 mL of hydrazine hydrate 50% aqueous solution was added to reduce the salts. The synthesized catalysts were separated by centrifugation and washed 3 times with H_2_O. A schematic representation of the preparation procedure is shown in Scheme S1. To check the reproducibility of the synthesis protocol, different batches of the same catalyst composition were obtained and tested catalytically. They all achieved the same activity (within experimental error).

Catalysts with various ratios of Au and/or Pt were prepared and denoted as sample Au_100%_@ZrO_2_, Pt_100%_@ZrO_2_, Au_85%_Pt_15%_@ZrO_2_, Au_75%_Pt_25%_@ZrO_2_, Au_67%_Pt_33%_@ZrO_2_, and Au_50%_Pt_50%_@ZrO_2_, respectively, according to the percentual metal content.

The catalytic tests were performed at 100 °C and 30 bar_g_ O_2_ pressure. First, zirconia support without metal loading was tested, and no reactivity by support alone was observed. Next, the tests were carried out in six parallel 75 mL stainless steel batch reactors (Parr 5000 series), with a typical load of 750 mg of catalysts and 40 mL of 0.25 M aqueous solution of Glc or GA as in the previously published paper.^20^

To determine the extent of Glc oxidation, a Thermo-Fisher Scientific UltiMateTM 3000 UHPLC instrument with the Rezex RHM-Monosaccharide H+ column with a guard column of the same type was used. Acidic reaction products were determined by using a Dionex ICS 3000 ion chromatograph equipped with an eluent generator, a suppressor, and a conductivity detector. Samples were collected at predefined times during the reaction and refrigerated until dilution with Milli-Q water, filtration, and analysis. No detection of gaseous products was performed.

Selectivity for products was calculated based on the carbon atoms in the compound, also assuming that 100% is the sum of all detected substrates and products.

Details on carbon balance (CB) calculations can be found in the Supporting Information, ES1.

Powder X-ray diffraction (XRD) analysis was performed using an X’Pert PRO diffractometer (PANalytical, Almelo, The Netherlands) with a copper source (Cu Kα radiation) operating at 50 kV and a current of 30 mA. The diffraction angle (2θ) was scanned between 10° and 120°. Phase identification was done by X’Pert HighScore Plus software using the PDF5 database. Rietveld refinements were carried out using TOPAS V3 software.^33^

Scanning electron microscopy (SEM) images were acquired on a Hitachi S-4800 instrument equipped with a Nanotrace EDX detector. Samples were prepared on carbon tape. Transmission electron microscopy (TEM) and high-resolution transmission electron microscopy (HRTEM) were performed using a JEM-2100 Plus Electron Microscope (JEOL) for microstructure evaluation. TEM micrographs were acquired using an accelerating voltage of 200 kV. The size distributions of NPs were determined by measuring ca. 200 particles using the ImageJ processing software. For EDX mapping, the samples were prepared in absolute ethanol (EtOH) solvent with ultrasonication at 5 °C in a period of 15 min to avoid aggregation, followed by depositing a drop of diluted suspensions of NPs on a 300 mesh Cu-coated carbon TEM grid. The sample-coated TEM grids were dried under a vacuum for 15 h. The electron microscope equipped with an energy-dispersive spectrometer X-max 20 mm^2^ (EDS, Oxford Instruments, High Wycombe, United Kingdom), enabling local analysis of elemental composition and acquisition of elemental distribution maps, was employed. EDX spectrum images were acquired on a Talos F200X TEM (Thermo Scientific, USA) equipped with a field emission gun operated at 200 kV, with 4 in-column SDD Super X detectors in the scanning TEM mode, with acquisition time 300s (10 frames, 30 s per frame) and resolution 400 × 400 pixels on an appropriate area (depending on the size of particles).

The surface area was determined using a 3Flex instrument (Micromeritics) by nitrogen adsorption at the boiling temperature of liquid nitrogen (77 K). Before adsorption experiments, all samples were degassed in two steps: 80 °C for 1 h and 300 °C for 12 h. The BET theory was used to determine the total surface areas.

The chemical nature of the catalysts was characterized by X-ray photoelectron spectroscopy (XPS) using a VG ESCA3MkII instrument at a base pressure better than 10^–9^ mbar, utilizing Al Kα radiation and a hemispherical analyzer operated at a constant pass energy of 20 eV. The spectra were calibrated by setting the main C 1 s component to a binding energy of 285 eV. The atomic composition of the catalyst surface was accomplished by assuming a homogeneous distribution of atoms and Scofield photoionization cross sections.

To better understand the structure and composition of bimetallic AuPt catalysts, density functional theory (DFT) calculations were carried out with VASP. Due to its favorable price-performance ratio, the revised PBE functional (RPBE)^34^ was used in the projector augmented wave (PAW) method.^19^ An energy cutoff of 500 eV sufficed for converged results, along with a Fermi smearing of 0.10 eV. For bulk structures, consisting of 32 atoms in a supercell, a dense 8 × 8 × 8 K-point mesh was used with a tight force convergence criterion of 0.01 eV/Å. Since ZrO_2_ itself was shown to be inactive (see Table 1), it was not modeled explicitly. Furthermore, the average metallic nanoparticles ranged from 7.5 to 11.9 nm, as shown by TEM micrographs (see Section 3.2.2 Morphology), which is large enough to warrant their description as metals. Thus, modeling focused on the formation of core–shell structures, adlayer formations, and miscibility, which did not require accounting for ZrO_2_. For pure Pt and Au in the fcc arrangement, experimentally known lattice constants were used as initial approximations and refined by varying the unit cell constant to arrive at 7.88 and 8.34 Å, respectively. For mixed compositions, full optimization of the unit cell was performed, which generally resulted in lower symmetry (triclinic) structures. For (111) surface slabs, four-layered structures with frozen bottom two layers and 20 Å of vacuum, along with the dipole corrections in the *z* direction, were used in 4 × 4 supercells, derived from the optimized bulk structures. Relative to the size of the supercell, a dense 3 × 3 × 1 Monkhorst–Pack K-point mesh was used. Since only surface atoms were substituted in this case, no unit cell optimization was performed, and only atom positions were allowed to change.

**Table 1:** Catalytic Results in the Oxidation of Glucose with Monometallic and Bimetallic Catalysts: Glc Concentration 0.25 M in Water, Temperature 100 °C, Pressure 30-Bar Gauge O_2_ for, and Glc to Metal Ratio 40:1^a^

catalysts	time period (h)	Glc conversion (%)	GO selectivity (%)	GA selectivity (%)	carbon balance (%)
Au_100%_ @ZrO_2_	3	100	57	5	67
Au_85%_Pt_15%_@ZrO_2_	3	100	55	23	100
Au_75%_Pt_25%_@ZrO_2_	3	100	52	34	95
Au_67%_Pt_33%_@ZrO_2_	3	100	44	44	100
Au_50%_Pt_50%_@ZrO_2_	3	100	53	20	76
Pt_100%_@ZrO_2_	3	18	76	12	96
ZrO_2_	3	0	0	0	

a Carbon balances were obtained using the ES1 in Supporting Information.

In bulk structures of 32 atoms in the fcc geometry, two regimes of substitution were investigated, which we view as two extreme cases. First, Pt (or Au) atoms are substituted by Au (or Pt) in such a way as to keep the same element atoms clustered together (denoted “adherent” henceforth). In the second regime, the atoms are substituted to achieve the maximum mixing or separations (denoted “disordered” henceforth). The mixing enthalpy is calculated as follows from eqs 1 and 2, where *E*_tot_ is the full DFT energy of the structure, *E*_bulk_^Au^ and *E*_bulk_^Pt^ are the energies of an Au or Pt atom, respectively, in bulk, and *E*_gas_^Au^ and *E*_gas_^Pt^ are their energies when isolated in a vacuum.

$$H_{\mathrm{mix}}=\frac{E_{\mathrm{tot}}-N_{\mathrm{Au}}E_{\mathrm{bulk}}^{\mathrm{Au}}-N_{\mathrm{Pt}}E_{\mathrm{bulk}}^{\mathrm{Pt}}}{N_{\mathrm{Au}}+N_{\mathrm{Pt}}}$$ 1

$$E_{\mathrm{cohesive}}=\frac{E_{\mathrm{tot}}-N_{\mathrm{Au}}E_{\mathrm{gas}}^{\mathrm{Au}}-N_{\mathrm{Pt}}E_{\mathrm{gas}}^{\mathrm{Pt}}}{N_{\mathrm{Au}}+N_{\mathrm{Pt}}}$$ 2

For slab structures, surface (top-layer) Pt (or Au) atoms are substituted with Au (or Pt). Similarly, the “adherent” and “disordered” configurations are studied. The *differential* substitution energy corresponds to the energy change, where one Pt atom from an infinite Pt(111) surface is used to substitute one Au atom in an already partially substituted Au(111)+*N* Pt structure, and vice versa. It is calculated as follows from eqs 3 and 4.

$$E_{\mathrm{substitution}(N\mathrm{Au}\to N\mathrm{Pt})}=E_{\mathrm{Au}(111)}^{N_{\mathrm{Au}}N_{\mathrm{Pt}}}+E_{\mathrm{bulk}}^{\mathrm{Au}}+\gamma_{111}^{\mathrm{Au}}-E_{\mathrm{Au}(111)}^{N+1_{\mathrm{Au}}N-1_{\mathrm{Pt}}}-E_{\mathrm{bulk}}^{\mathrm{Pt}}-\gamma_{111}^{\mathrm{Pt}}$$ 3

$$E_{\mathrm{substitution}(N\mathrm{Pt}\to N\mathrm{Au})}=E_{\mathrm{Pt}(111)}^{N_{\mathrm{Au}}N_{\mathrm{Pt}}}+E_{\mathrm{bulk}}^{\mathrm{Pt}}+\gamma_{111}^{\mathrm{Pt}}-E_{\mathrm{Au}(111)}^{N+1_{\mathrm{Pt}}N-1_{\mathrm{Au}}}-E_{\mathrm{bulk}}^{\mathrm{Au}}-\gamma_{111}^{\mathrm{Au}}$$ 4

## 3 Results

### 3.1 Catalytic Tests of Mono- and Bimetallic Catalysts

The performance of the Au, Pt, and AuPt catalysts in the oxidation of Glc is plotted in Figure 1. Most bimetallic catalysts reached full Glc conversion within ∼30 min. The main products observed were GO and GA, along with other side products such as GLU, TA, TO, GLY, FA, and OA. Initial tests with the monometallic catalyst (Figure 1a,f) showed that Au_100%_@ZrO_2_ achieved complete Glc conversion within 1.5 h. After 3 h (Table 1), the catalyst exhibited 57% selectivity toward GO as the main product and 5% toward GA, with a low CB indicating significant formation of side products from GA overoxidation.

**Figure 1:**
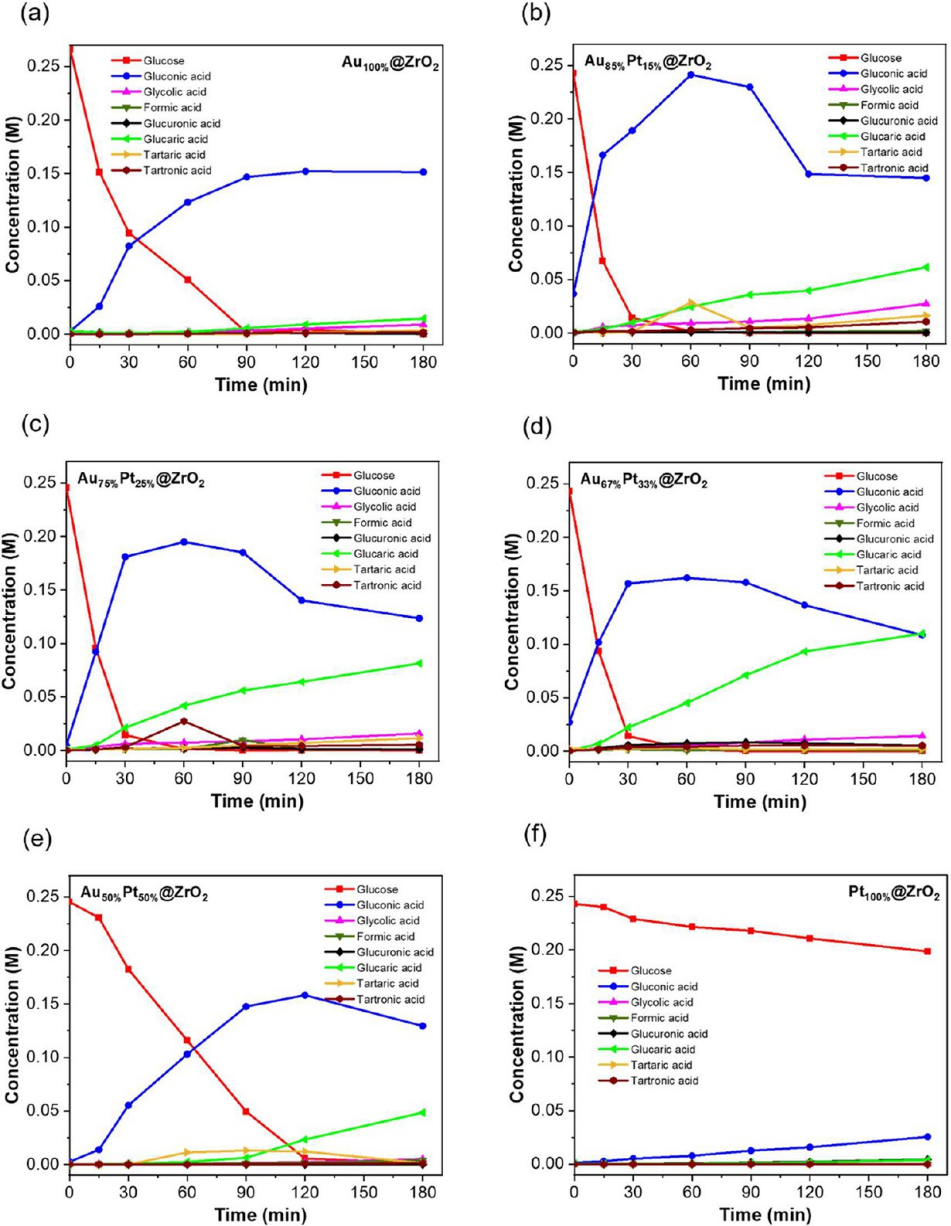
Concentration profiles of glucose on mono- and different bimetallic catalysts at conditions of 100 °C, 30-bar gauge O_2_ pressure, 0.25 M glucose concentration, a metal ratio of 40:1, and a reaction time of 3 h for different catalyst composition: (a) Au_100%_@ZrO_2_, (b) Au_85%_Pt_15%_@ZrO_2_, (c) Au_75%_Pt_25%_@ZrO_2_, (d) Au_67%_Pt_33%_@ZrO_2_, (e) Au_50%_Pt_50%_@ZrO_2_, and (f) Pt_100%_@ZrO_2_. Reaction products were determined by means of ion chromatography.

To improve GA selectivity, the Au/Pt ratio was systematically varied. The introduction of Pt markedly enhanced the catalytic activity (Figure 1b–e).

As shown in Figure 1b and Table 1, Au_85%_Pt_15%_@ZrO_2_ exhibited 93% GO selectivity after 15 min, which gradually decreased over time as GA selectivity increased, reaching 23% after 3 h. A similar trend occurred for Au_75%_Pt_25%_@ZrO_2_ and Au_67%_Pt_33%_@ZrO_2_, with final selectivities of 52% for GO, 34% for GA, and 44% for GO and 44% for GA, respectively. Increasing the Pt content up to an equal Au/Pt ratio (Au_50%_Pt_50%_@ZrO_2_; Figure 1e and Table 1) led to a lower final selectivity of 53% for GO and 20% for GA, while Pt_100%_@ZrO_2_ showed an even greater loss of activity, with only 15% GO and 2% GA (Figure 1f and Table 1). As mentioned above, monometallic Au efficiently catalyzed the initial oxidation of Glc to GO but showed limited activity for GO-to-GA conversion. In contrast, bimetallic catalysts facilitated this second oxidation step more effectively, indicating higher overall oxidation activity for AuPt compared to monometallic Au or Pt.

Varying the Au:Pt ratio has a significant effect, showing an almost doubled selectivity toward GA when changing from a 50:50 to 67:33 ratio. The performance of the best catalyst—Au_67_%Pt_33_%@ZrO_2_ is comparable with various catalysts reported in the literature, with an emphasis on the production of GA from Glc under base-free conditions presented in Table S2 in the Supporting Information. It is also important to note that the AuPt catalysts used in this work showed catalytic activity in a lower oxygen pressure range and reached full conversion of Glc within a shorter time, less than 30 min.

The CB was determined by adding up the carbon amounts of the HPLC-detected molecules present in the aqueous phase to observe trends in the broad spectrum of products obtained during the reaction. The calculated percentage values are gathered in Table 1. The lowest CB value was reached for the monometallic Au_100_%@ZrO_2_ catalyst and Au_50_%Pt_50_%@ZrO_2_, 67% and 76% respectively, which suggests their high potential to overoxidize glucose and form volatile carbon-containing products, most likely CO_2_. For all remaining catalysts, CB values do not drop below 95%, pointing to a more efficient conversion of glucose into value-added compounds, underlying the superiority of the bimetallic catalysts. Other side products, such as polymers, e.g., humins, can also form; however, they cannot be detected with the applied detection method. Change of pH as a function of time was also measured during the reaction. The initial pH was around 6 in an aqueous solution of Glc and decreased rapidly to 2.5, confirming the formation of (di)­carboxylic acids. These results can be explained by theoretical calculations. As shown previously,^20^ the oxidation of glucose to GO with O* or O_2_* is faster on Au (barriers of 0.24 and 0.21 eV, respectively) than on Pt (0.57 and 0.50 eV, respectively). At higher temperatures, even though C–C bond cleavage is thermodynamically favorable on Pt (−0.06 eV), it is nonselective. In contrast, on Au (+1.40 eV), cleavage does not occur at all.

In order to better understand the mechanistic aspect of the mono- and bimetallic catalysts, controlled experiments were also performed with GO as a substrate; see Supporting Information Figure S1. When GO was employed as the starting reactant, the experiment revealed that monometallic catalysts, Au_100%_@ZrO_2_ and Pt_100%_@ZrO_2_, did not show any activity for the formation of GA (Figure S1a,f). For bimetallic catalysts, GA was the main product in the reaction mixture with selectivity of 26, 23, 23, and 56%, respectively (Figure S1b–e), and GLY was a minor side product. This suggests that for the conversion of GO, a bimetallic catalyst is essential. Furthermore, GA continued to react to form GLY, which is a product of C–C bond cleavage (Scheme 1).

As seen from our study of Glc concentration–time profiles, Glc oxidation mainly produces oxidized products with shorter carbon chains, such as TA (C4), TO (C3), GLY (C2), OX (C2), and FA (C1). Based on the literature results and our initial study, it is seen that the oxidation of Glc involves a series of complex parallel and consecutive reactions in the presence of monometallic (Au, Pt) and bimetallic (AuPt) catalysts on ZrO_2_ support, as both C–C and C–H cleavage occur at different positions on the C6 carbon chain.

### 3.2 Characterization of the Catalysts

#### 3.2.1 Crystallographic Structure and Composition

XRD measurements were performed to confirm the introduction of Au and/or Pt NPs with different ratios and the crystallographic phase of the ZrO_2_ support. The diffraction patterns of the samples under study are shown in Figure S2 in the Supporting Information, alongside the diffractogram of pure ZrO_2_ for a better comparison. Only the region where the individual (not overlapped with ZrO_2_) Au and/or Pt peaks are visible is shown, with vertical lines and arrows marking Au and Pt reflections, respectively. In the case of the pure Au (red) and Pt (violet) samples, the additional peaks corresponding to these phases can be distinguished, with the gold peaks being notably narrower, indicating a significantly larger crystallite size compared to Pt. In the bimetallic samples, individual Pt peaks are not observed; instead, only a shoulder appears on the left side of the first two Au peaks. Phase identification confirmed the presence of monoclinic (*P21/C*) ZrO_2_ in all synthesized samples. In the case of monometallic samples Au_100%_@ZrO_2_ and Pt_100%_@ZrO_2_, pure Au (*Fm3̅m*) and Pt (*Fm3̅m*) phases were detected, respectively. The refined lattice parameter and refined volume-weighted mean crystallite size (L_Vol_-IB) are *a* (Au): 4.078 Å, L_Vol_-IB­(Au): 32 nm, and *a* (Pt): 3.920 Å, L_Vol_-IB­(Pt): 8 nm. However, the absence of well-defined Au/Pt peaks in the bimetallic samples (see the left broadening at ∼38° and ∼44° in Figure S2), along with the fact that both Pt and Au have the same structure and space group with similar lattice parameters, as well as the overlap with the ZrO_2_ peaks, makes quantitative analysis challenging. Rietveld refinements yielded good results for all the bimetallic samples (*R*_wp_ ∼ 5%) using a single cubic Au-based (*Fm3̅m*) phase, with a refined lattice parameter smaller than that of pure Au, e.g., 4.038Å for Au_67%_Pt_33%_@ZrO_2_, suggesting the formation of an alloy with a lattice parameter between those of Pt and Au. The use of two phases, one Au-based and the other Pt-based phases, did not improve the refinement. Due to the lack of well-defined peaks, it is not possible to determine whether there is only one or several AuPt phases using this technique. Consequently, the samples were analyzed using TEM for further investigation.

The elemental composition of the catalysts was determined using SEM-EDX measurements and confirmed by XPS (gathered in Tables S3 and S6 in the Supporting Information). The observed atomic percentages of Au and Pt for Au_85%_Pt_15%_@ZrO_2_, Au_75%_Pt_25%_@ZrO_2_, Au_67%_Pt_33%_@ZrO_2_, and Au_50%_Pt_50%_@ZrO_2_ were found to be 85:12, 80:20, 57:43, and 50:43, respectively. These values closely agree with the theoretically calculated atomic percentages, demonstrating the consistency between expected and experimental compositions.

#### 3.2.2 Morphology

SEM images of Au, Pt, and Au/Pt nanoparticles on ZrO_2_ support are gathered in Figure S3 in the Supporting Information, in which individual Au and Pt NPs in monometallic samples are visible on the ZrO_2_ surface (marked with circles). In the case of the bimetallic catalysts with different Au/Pt ratios (Figure S7b–e), NPs are found to be distributed uniformly on the support material.

TEM micrographs of monometallic and bimetallic samples are presented in Figure 2. The ZrO_2_ support is coated with metal Au and Pt NPs that can be distinguished by their contrast (appearing “darker” compared to that of ZrO_2_). The size of the pure monometallic Au NPs on top of the ZrO_2_ support (Au_100%_@ZrO_2_) was assessed to be 15 nm, as shown in Figure 2a, while the average size of Pt is 100 nm, as shown in Figure 2f. For bimetallic samples, the Au/Pt NPs are mostly spherically shaped, with a smaller size distribution when compared to individual Au and Pt nanoparticles in monometallic samples; see Figure 2b–e. The average size of Au/Pt NPs was observed as 11.9 nm in Au_85%_Pt_15%_@ZrO_2_, 10.3 nm in Au_75%_Pt_25%_@ZrO_2_, 8.6 nm in Au_67%_Pt_33%_@ZrO_2_, and 7.5 nm in Au_50%_Pt_50%_@ZrO_2_. For details on size distribution, see Figure S4. TEM images in Figure 2a–f also suggest the poor porosity of the ZrO_2_ support, as a majority of metallic particles exist at the top of the ZrO_2_ support surface.

**Figure 2:**
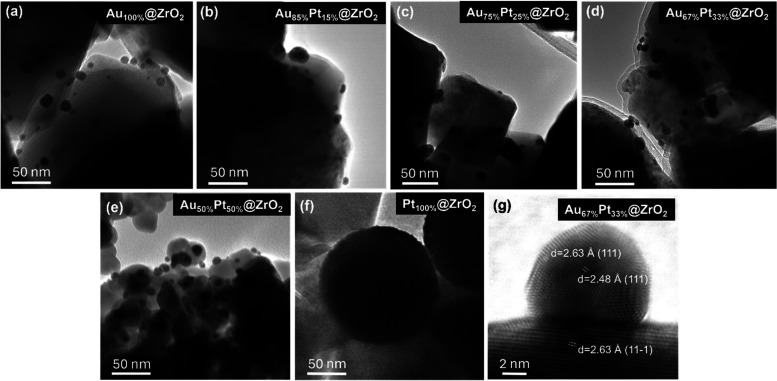
TEM micrographs of samples (a) Au_100%_@ZrO_2_, (b) Au_85%_Pt_15%_@ZrO_2_, (c) Au_75%_Pt_25%_@ZrO_2_, (d) Au_67%_Pt_33%_@ZrO_2_, (e) Au_50%_Pt_50%_@ZrO_2,_ (f) Pt_100%_@ZrO_2_, and (g) HR-TEM of Au_67%_Pt_33%_@ZrO_2_.

In the HR-TEM image of Au_67%_Pt_33%_@ZrO_2_ (Figure 2g), the lattice fringes were observed, and the determined interplanar spacing *d* = 2.63 Å corresponds to the (111) plane of Au, and *d* = 2.48 Å corresponds to the (111) plane of Pt, whereas *d* = 2.24 Å corresponds to the (11–1) plane of ZrO_2_. This is in line with the existing literature, in which it was found that the precursors of Pt and Au were rapidly reduced into metallic atoms by adding hydrazine hydrate due to its strong reducing properties, leading to the immediate formation of metallic atoms and keeping the particles relatively small in comparison to the monometallic counterparts.

EDX spectroscopy was carried out with a high-angle annular dark-field scanning transmission electron microscope (HAADF-STEM) mode in order to check the morphology of bimetallic nanoparticles with changing Au/Pt ratios. In particular, elemental mapping was recorded on selected nanoparticles where both Au and Pt signals were collected and then presented with different colors, as shown in Figures 3–6.

**Figure 3:**
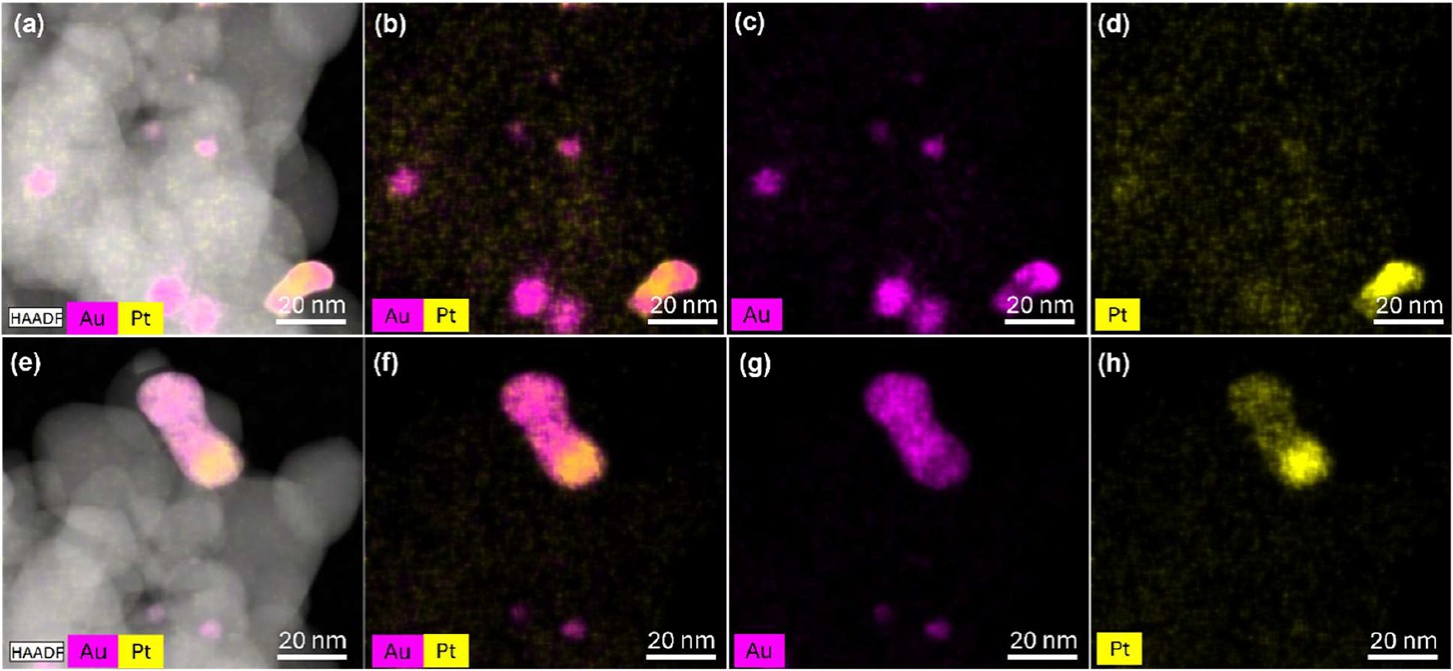
High-angle annular dark field scanning transmission electron microscopy (HAADF-STEM) images in two different selected areas and corresponding elemental mapping of the Au_85%_Pt_15%_@ZrO_2_ bimetallic catalyst. (a,e) HAADF-STEM images of the selected area show the morphology of the nanoparticles. (b,f) Mixed elemental distribution map of Au and Pt; (c,g) elemental distribution of Au; (d,h) elemental distribution of Pt.

The elemental distribution on the bimetallic sample with the highest amount of Au, Au_85_%Pt_15_%@ZrO_2_ (Figure 3), shows that the two elements coexist with each other in the scanned region in the form of scattered small particles as well as larger particles with a slight tendency for segregation of metals in the form of Janus-type particles. For the latter, it can be observed that some regions are enriched in one of the metals, yet still create an alloy of Au and Pt. For the bimetallic samples Au_75_%Pt_25_%@ZrO_2_ and Au_67_%Pt_33_%@ZrO_2_, a much higher level of intermixing of gold and platinum components is observed with the visible formation of alloys and core–shell structures, as shown with TEM images with elemental mapping (Figures 4 and 5, respectively). While for Au_75_%Pt_25_%@ZrO_2_ the Pt shell is relatively thin and dense, for Au_67_%Pt_33_%@ZrO_2_ intermixing is observed even within the shell border, where the Au presence is enriched. Moreover, the bimetallic particles feature higher dispersion, with particle sizes smaller than in the case of the monometallic ones.

**Figure 4:**
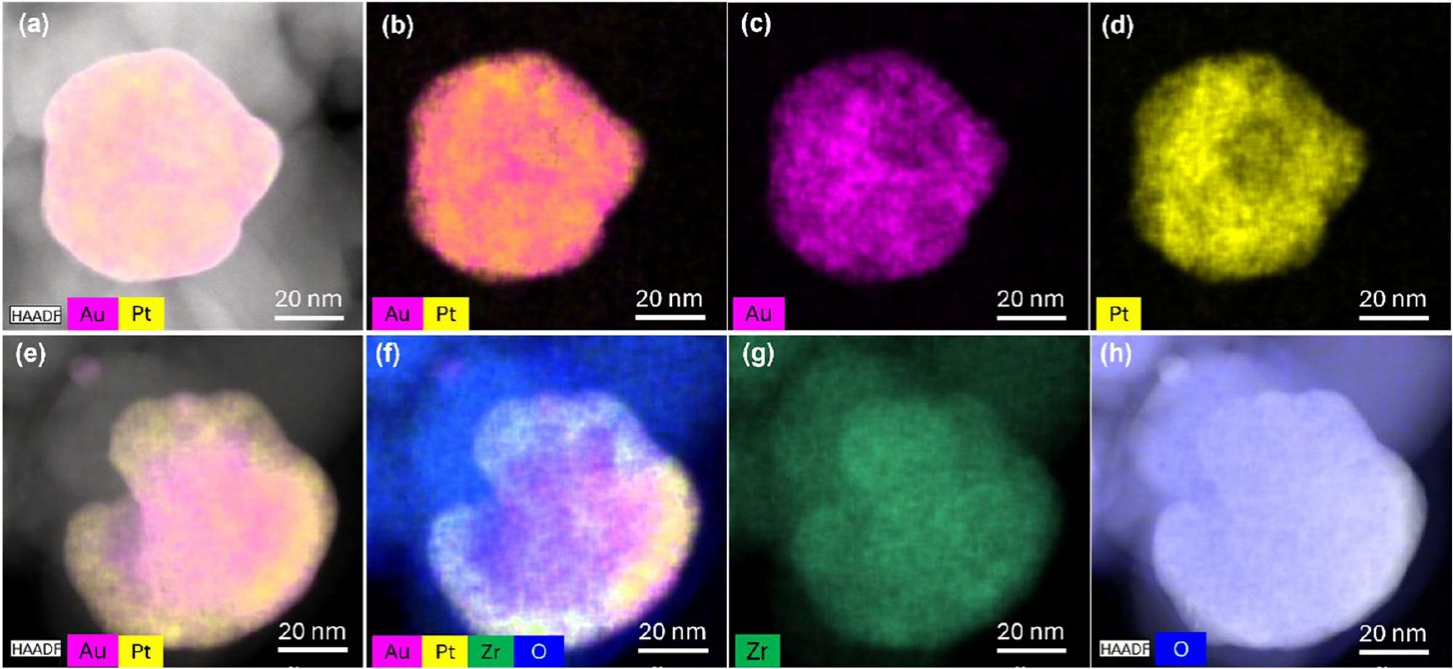
High-angle annular dark field scanning transmission electron microscopy (HAADF-STEM) images in two selected areas (top and bottom panel) and corresponding elemental mapping of the Au_75%_Pt_25%_@ZrO_2_ bimetallic catalyst. (a,e) HAADF-STEM images of the selected area show the morphology of the nanoparticles, (b) mixed elemental distribution map of Au and Pt, (c) elemental distribution of Au, (d) elemental distribution of Pt, (f) elemental distribution of Au, Pt, Zr, and O; (g) elemental distribution of Zr and (h) elemental distribution of O.

**Figure 5:**
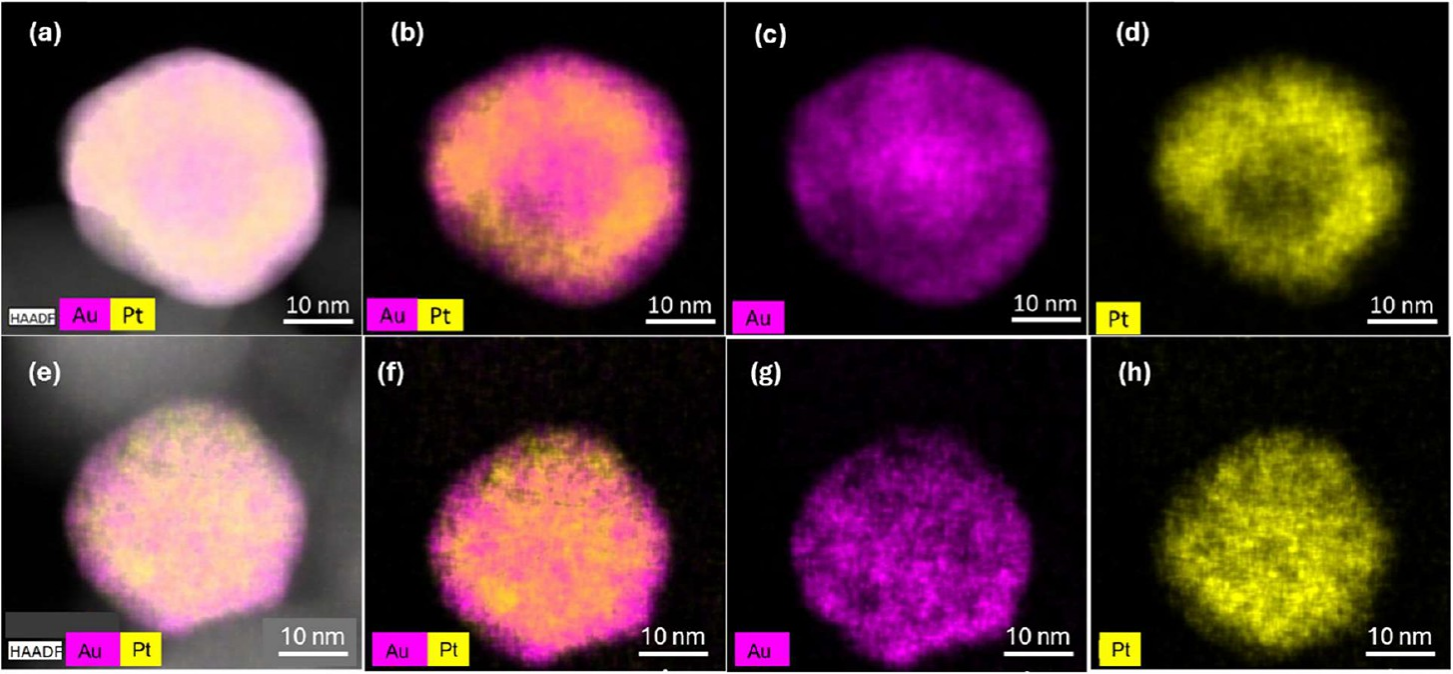
HAADF-STEM images in two selected areas (top and bottom panel) and corresponding elemental mapping of the Au_67%_Pt_33%_@ZrO_2_ bimetallic catalyst. (a,e) HAADF-STEM images showing the morphology of the nanoparticles. (b,f) Mixed elemental distribution map of Au and Pt, (c,g) elemental distribution of Au, and (d,h) elemental distribution of Pt.

In the last studied bimetallic catalyst where an equal ratio of Au and Pt is present, Au_50%_Pt_50%_@ZrO_2_, see Figure 6, the metals tend to segregate in the way that Pt appears in the form of very small and clustered particles and Au rather groups in agglomerates. This is reflected in a wide distribution of the particle sizes for this catalyst; see Figure S4 in the Supporting Information.

**Figure 6:**
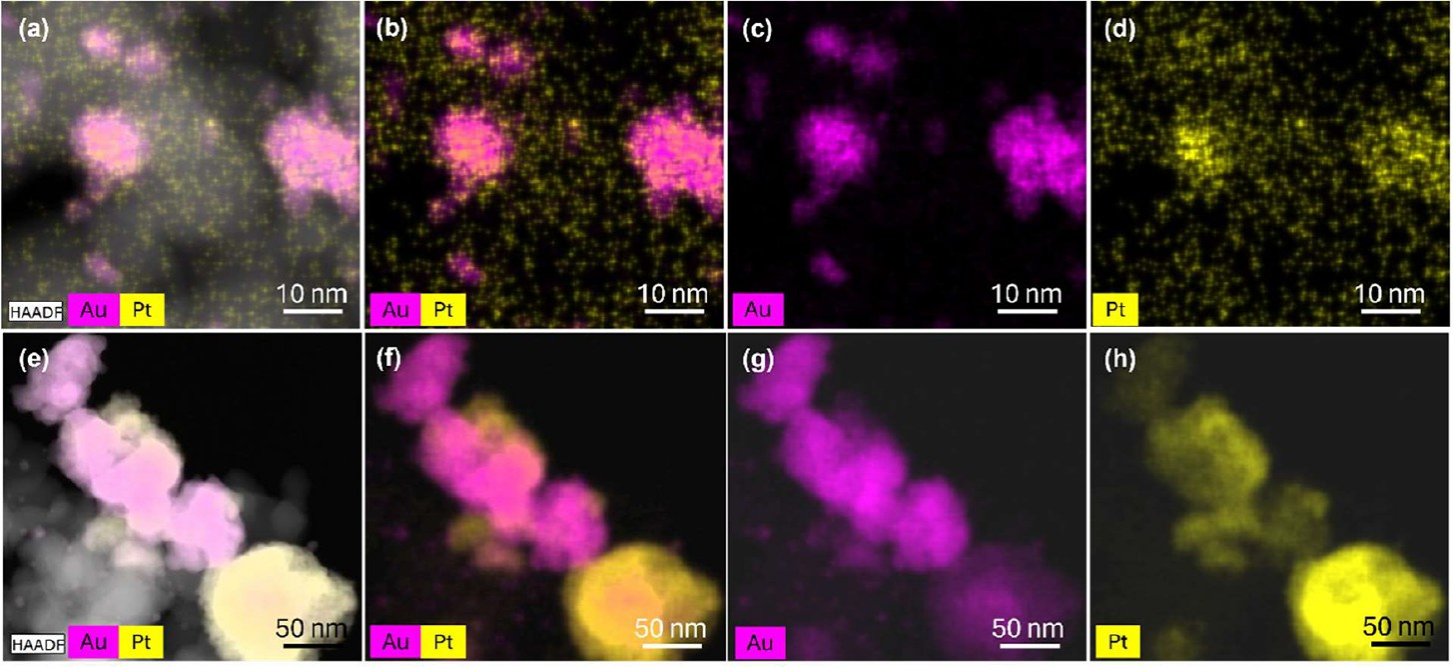
HAADF-STEM images in two different selected areas and corresponding elemental mapping of the Au_50%_Pt_50%_@ZrO_2_ bimetallic catalyst. (a,e) HAADF-STEM images of the selected area showing the morphology of the nanoparticles. (b,f) Mixed elemental distribution map of Au and Pt, (c,g) elemental distribution of Au, and (d,h) elemental distribution of Pt.

#### 3.2.3 Surface Area and Porosity

The surface area is a crucial parameter for the adsorption of Glc as the reaction occurs on the well-distributed active sites in the form of NPs or the NP alloys present on the ZrO_2_ support. Surface area analysis for monometallic and bimetallic catalyst samples was carried out using the BET method from the nitrogen adsorption–desorption isotherms. The estimated values of the specific surface area and mean pore diameter of mono- and bimetallic catalysts are summarized in Table S5 in the Supporting Information. From these data, it is visible that the surface area of bimetallic catalysts slightly increases with rising Pt concentration in the support, while the lowest surface areas characterize monometallic samples. This is possible due to the higher agglomeration of the particles, which was observed in the SEM and TEM micrographs. The input in support of the overall porosity of the samples is low, as the predominant support material, ZrO_2,_ exhibits a low surface area and particle size of around 1 μm.

## 4 Discussion

### 4.1 Composition vs Activity

For most noble metals, the interaction between active centers and carboxylic groups is weak, while C–C bond activation is significant, leading to straightforward decarboxylation on surfaces like Pt and Au.^10,29,32,35^ Theoretical modeling presented in our recent study^20^ predicted that Pt could stand out as a superior catalyst component in comparison with Au and Cu. The same study also showed that pure Pt does not produce GA, but rather undesired short-chain products.

In this work, the comparison of the monometallic and bimetallic Au and Pt catalysts confirmed the previous predictions of Pt being the necessary component of the catalyst. Materials with variable Au/Pt ratios indeed enabled the direct production of GA from glucose in the sought-after one-pot reaction from Glc involving GO as an intermediate, contrary to the monometallic catalysts, which dominantly produce GO, as shown in Figure 1. The varied compositions in bimetallic catalysts were accompanied by a change in the particle size, seen by TEM analysis, with particle size evolving from 11.9 nm for the Au_85%_Pt_15%_@ZrO_2_ to 4.8 nm for the Au_50%_Pt_50%_@ZrO_2_ catalyst.

The peak production of the GO intermediate increases with Au content for bimetallic catalysts, as shown in Figure 1b, reaching a maximum for the monometallic Au_85%_Pt_15%_@ZrO_2_ catalyst, while the efficiency of GA formation does not follow this trend. For reaching an optimal synergistic effect of Au and Pt, a 1:1 Au/Pt ratio of the metals should be achieved, possibly offering the highest vicinity of the Au and Pt sites for leveraging synergistic effects in the two reaction steps involved.

Correlating experimentally obtained compositions of the catalyst with the TEM-EDX mapping reveals that the Au/Pt ratio influences intermixing, which yields a spectrum of structures, including intermixed alloys, core–shell, and segregated monometallic particles, which coexist. The imaging of Au_85%_Pt_15%_@ZrO_2_, and Au_50%_Pt_50%_@ZrO_2_ catalysts shows the highest level of segregation of the two metals, including the formation of Janus-type particles, while in the Au_75%_Pt_25%_@ZrO_2_ catalyst, with dominant intermixed alloy and core–shell particles, the latter with an Au core and a thin Pt shell. The best-performing GA-producing catalyst, Au_67%_Pt_33%_@ZrO_2_, possesses alloy and core–shell structures and additionally exhibits smaller particle sizes than Au_75%_Pt_25%_@ZrO_2,_ enhancing surface-to-bulk ratio, ergo availability of the active sites. The XRD also confirmed alloy formation in the bimetallic catalysts.

The metal ratio affects the catalyst performance through its effects on the particle morphology. The superior performance of Au_67_%Pt_33_%@ZrO_2_ is ascribed to its ability to form intermixed alloy and core–shell particles. As shown later in the modeling part, highly intermixed alloys are thermodynamically unfavorable and must therefore form due to kinetic considerations during catalyst synthesis. Au_85_%Pt_15_%@ZrO_2_ and Au_50_%Pt_50_%@ZrO_2_, where segregation was observed, performed noticeably poorer. Thus, Au and Pt represent a superior tandem for glucose oxidation to GA due to the electronic properties of their alloys and core–shell structure, while the precise ratio of the metals is required to achieve sufficient mixing.

### 4.2 Computational Insight into Alloying

A large number of Pt and Au nuclei form instantaneously during the synthesis, which provides a suitable template for the homogeneous crystal growth of both monometallic nanoparticles. From a kinetics perspective, the mechanism for electron transfer from Au^3+^ to Pt^4+^ is provided by ZrO_2_, which helps in the coreduction of Au and Pt ions.^36^ It is important to note that the presence of the Au–Pt interaction in solution allows AuPt alloys to potentially nucleate at the atomic level. Thus, the newly formed Au and Pt atoms quickly fuse together to form small AuPt nuclei. The strong metal–metal interaction will lead to changes in the structure of the catalyst interface, and the stabilization of transition states on alloy catalysts is an additional benefit.^36^ In addition, the slight difference in electronegativity between Pt and Au could also intrinsically affect the final morphology. A slightly higher electron affinity of Au might lead to faster reduction rates in the aqueous solution. As a result, the bond length and electronic configuration of Pt–Pt sites might be altered by the Au–Au presence, which contributes to enhanced catalytic performances during the oxidation of Glc and GO. On the other hand, experimental characterization, especially TEM mapping, and previous computational studies show that these metals tend to segregate.^37^ The enthalpy of mixing was determined by using DFT calculations to elucidate the stability of the alloys. It was discovered that enthalpy remains positive (unfavorable) for all compositions, as shown in Figure 7a. This confirms that thermodynamically, the segregation is favored. Since the reaction proceeds at a relatively high temperature of 100 °C, where the average kinetic energy per atom is 0.05 eV, the thermodynamic difference of a few hundred eV is easily overcome. Consequently, segregation readily occurs.

**Figure 7:**
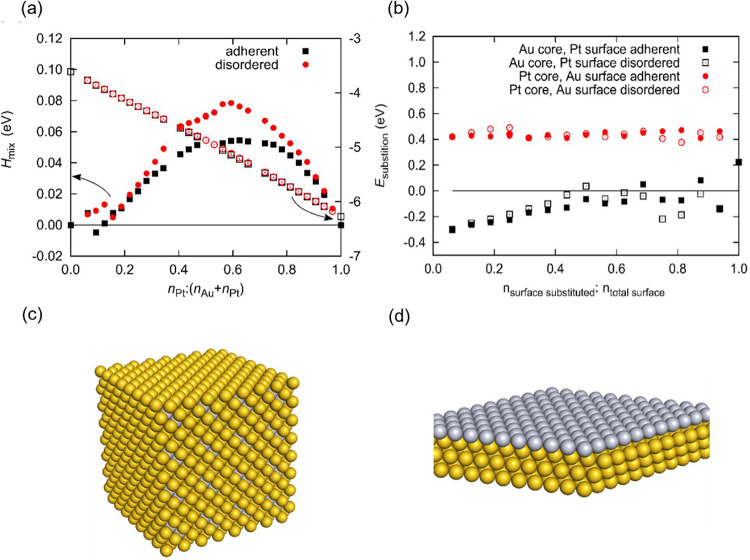
(a) DFT-calculated enthalpy of mixing (full symbols) and cohesive energies (empty symbols) for different Au/Pt ratios, (b) DFT-calculated *differential* enthalpy of substitution on Au(111) and Pt(111) surfaces, (c) a model of Au_30_Pt_2_ alloy, and (d) Au-core/Pt-surface core–shell structure.

The enthalpy of mixing increases more quickly when Au atoms are introduced into bulk Pt than the other way around. Low Pt/Au ratio mixtures are only slightly unfavorable and might even have a negative mixing enthalpy (at an atomic ratio of 2:30), although the effect is smaller than the accuracy of the DFT method. Thus, we expect to observe clustered Pt particles and Au agglomerates, which might have small amounts of Pt. This explains the experimental data, where better mixing and alloying are observed in high-Au/low-Pt catalysts. Using TEM, we observed that Au/Pt NPs have a narrower size distribution compared with monometallic particles. Small particles have different energetics than bulk systems, and our DFT treatment accounted for the latter. It was also observed that the samples with the lowest Pt content (Au_85_%Pt_15_%@ZrO_2_) coexisted as alloys, while in other samples, core–shell structures were also observed. Of those, structures with Au are located in the core form. This is explained by the DFT calculations, which show a negative (favorable) *E*_(substitution­(*N* Au → *N* Pt)) and a positive (unfavorable) *E*_(substitution­(*N* Pt → *N* Au)) with little concentration dependence (Figure 7b). In essence, this means that a core–shell structure with Au in the core with a surface Pt is favorable, while the opposite is not valid. Noting that the DFT calculations were performed in the plane-wave formalism, which supposes periodic boundary conditions, the results are more relevant for larger, bulk-like structures; see the used models in Figure 7c,d. Of those, core–shell structures are predicted to be more stable than alloys, while NPs also form alloys, as experimentally shown.

## 5 Conclusion

In this work, zirconia-supported monometallic Au, Pt, and bimetallic AuPt catalysts with variable composition were investigated in the aerobic direct oxidation of glucose to GA. The best-performing catalyst, yielding 44% GA selectivity, turned out to be the one with a composition of 67% and 33% of Au and Pt, respectively. This catalyst, primarily composed of alloyed particles, was confirmed by accompanying computations for compositions with a prevailing gold fraction. This study demonstrates that high-fidelity control of catalyst performance can be achieved in the direct oxidation of glucose to GA using composition-optimized bimetallic nanocatalysts dispersed on a suitable support.

## Supplementary Material



## Notes

Additional information on details on catalyst preparation, characterization and catalytic activity (PDF)
