# Supplementary material for: Direct Oxidation of Glucose to Glucaric Acid Using Bimetallic AuPt/ZrO2 Nanocatalysts
Source: ACS Appl Nano Mater. 2025 Oct 23;8(44):21370–81. doi: 10.1021/acsanm.5c03743 (PMC12603993; doi:10.1021/acsanm.5c03743)
Supplement: Supplementary file 1 [file an5c03743_si_001.pdf]

Supporting Information

Direct Oxidation of Glucose to Glucaric acid using Bimetallic AuPt/ZrO_2 Nanocatalysts

Joanna Elzbieta Olszowka,[1] Abdul Selim,[1] Žan Lavrič,[2,3] Janvit Teržan,[2] Ana Kroflič,[2] Miha Grilc,[2] Blaž Likozar,[2] Jaroslav Kupčík,[4,5] Esther de Prado,[4] Jan Plsek,[6] Eliska Mikyskova,[7] Jaroslava Moravkova,[8] Matej Huš,[2,9,10] Stefan Vajda[1]*

Corresponding author's email address: joanna.olszowka@jh-inst.cas.cz

[1] Department of Nanocatalysis, J. Heyrovský Institute of Physical Chemistry of the CAS, Dolejškova 2155/3, 182 23, Prague, Czech Republic
E-mail: joanna.olszowka@jh-inst.cas.cz
[2] Department of Catalysis and Chemical Reaction Engineering, National Institute of Chemistry, Hajdrihova 19, 1000 Ljubljana, Slovenia
[3] University of Nova Gorica, Vipavska 13, SI-5000 Nova Gorica, Slovenia
[4] Department of Material Analysis, Institute of Physics of the CAS, Na Slovance 1999/2, 182 00, Prague, Czech Republic
[5] Centre of Instrumental Techniques (CIT), Institute of Inorganic Chemistry of the Czech Academy of Sciences, Husinec-Řež, 1001, 250 68 Husinec-Řež, Czech Republic
[6] Department of Low-dimensional Systems, J. Heyrovský Institute of Physical Chemistry of the CAS, Dolejškova 2155/3, 182 23, Prague, Czech Republic
[7] Center for Innovations in the Field of Nanomaterials and Nanotechnologies, J. Heyrovský Institute of Physical Chemistry of the CAS, Dolejškova 2155/3, 182 23, Prague, Czech Republic
[8] Department of Structure and Dynamics in Catalysis, J. Heyrovský Institute of Physical Chemistry of the CAS, Dolejškova 2155/3, 182 23, Prague, Czech Republic
[9] Association for Technical Culture of Slovenia (ZOTKS), Zaloška 65, SI-1000 Ljubljana, Slovenia
[10] Institute for the Protection of Cultural Heritage of Slovenia (ZVKDS), Poljanska 40, SI-1000 Ljubljana, Slovenia

Details on sample preparation.

Table S1. Synthesis of mono and bimetallic catalysts with variable Au and Pt content.

Catalyst	Au/Pt content	Reagents used		
	(%)	$HAuCl_4 \cdot 3H_2O$ (mmol)	$H_2PtCl_6 \cdot xH_2O$ (mmol)	NH_2-NH_2 $.H_2O$ (mmol)
$Au_{100\%}$@ZrO_2	100:00	1.65	--	0.024
$Au_{85\%}Pt_{15\%}$@ZrO_2	85:15	1.35	0.24	0.024
$Au_{75\%}Pt_{25\%}$@ZrO_2	75:25	1.19	0.40	0.024
$Au_{67\%}Pt_{33\%}$@ZrO_2	67:33	1.07	0.53	0.024
$Au_{50\%}Pt_{50\%}$@ZrO_2	50:50	0.79	0.80	0.024
$Pt_{100\%}$@ZrO_2	00:100	-	1.61	0.024

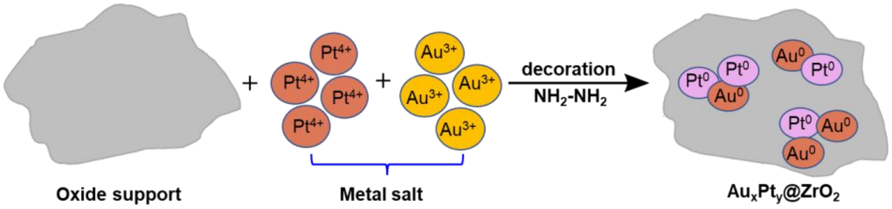

Scheme S1. Schematic presentation of the mono- and bimetallic catalysts synthesis.

Catalytic activity

Table S2. Comparison between catalytic performance and reaction conditions for the production of glucaric acid from glucose reported in the literature.

Catalyst	pO_2 (bar)	Temp (°C)	Glucose (mol/L)	Time (h)	Conv. (%)	Yield (%)	Sel. (%)	Ref.
$PtCu/TiO_2$	1	45	0.56	4	100	-	32.3	[1]
Pt_1Cu_3/TiO_2	1	45	0.56	6	100	-	9.3	[1]
1%Pt 1%Pd/TiO_2	1	45	0.8	10	100	37	37	[2]
5%Pt/C	13.8	80	0.61	10	99	74	73	[3]
1%Au/AC	10	60	0.328	3	100	25	25	[4]
3.5%Au 3.5%Pt/ZrO_2	40	100	0.25	2	100	50	50	[5]
Au/AC	10	60	0.29	1	88	25	22	[6]
5%Pt/CNT	10	60	0.025	4	100	82	82	[7]
Au/Ti-NT	6	80	0.25	6	73	-	18.5	[8]
$Au_{15}Pd_{85}$/Ti-NT	6	80	0.25	6	64	-	1.7	[8]
4%Pt 1.7% Cu/TiO_2	15	90	0.278	12	92	60	55.2	[9]
Au−Pt/ZrO_2	40	100	0.25	8	100	71	71	[10]
**$Au_{67\%}Pt_{33\%}@ZrO_2$**	**30**	**100**	**0.25**	**3**	**100**	**44**	**44**	**This study**

Details on carbon balance calculations.

Carbon balance (CB %) is defined as the ratio of the total amounts of carbon in the products formed to the existing total carbon in converted reactants initially and calculated as in the following equation S1.

$$CB\ (\%) = \frac{\text{n of products and unreacted Glc}*\text{carbon atoms in the given molecule}}{\text{initial n of Glc}*6} \qquad \text{(ES1)}$$

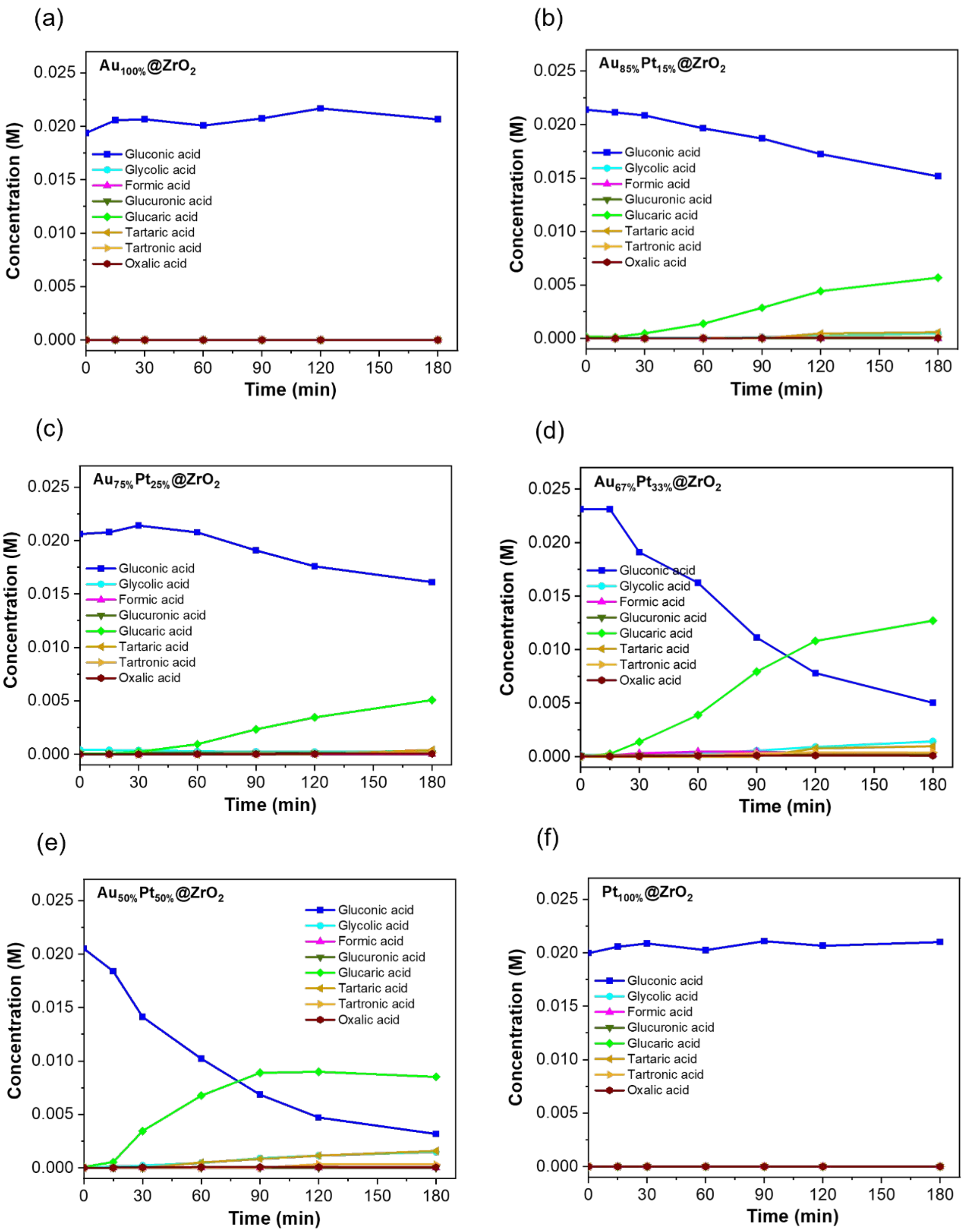

Figure S1. Catalyst activity in the oxidation of gluconic acid to glucaric acid. Concentration profiles for gluconic acid oxidation over (a) $Au_{100\%}@ZrO_2$, (b) $Au_{85\%}Pt_{15\%}@ZrO_2$, (c) $Au_{75\%}Pt_{25\%}@ZrO_2$, (d) $Au_{67\%}Pt_{33\%}@ZrO_2$, (e) $Au_{50\%}Pt_{50\%}@ZrO_2$ at 100 °C, 30 bar g O_2, approximate initial gluconic acid concentration of 0.020 M, and reaction time of 3 h.

XRD analysis of the samples.

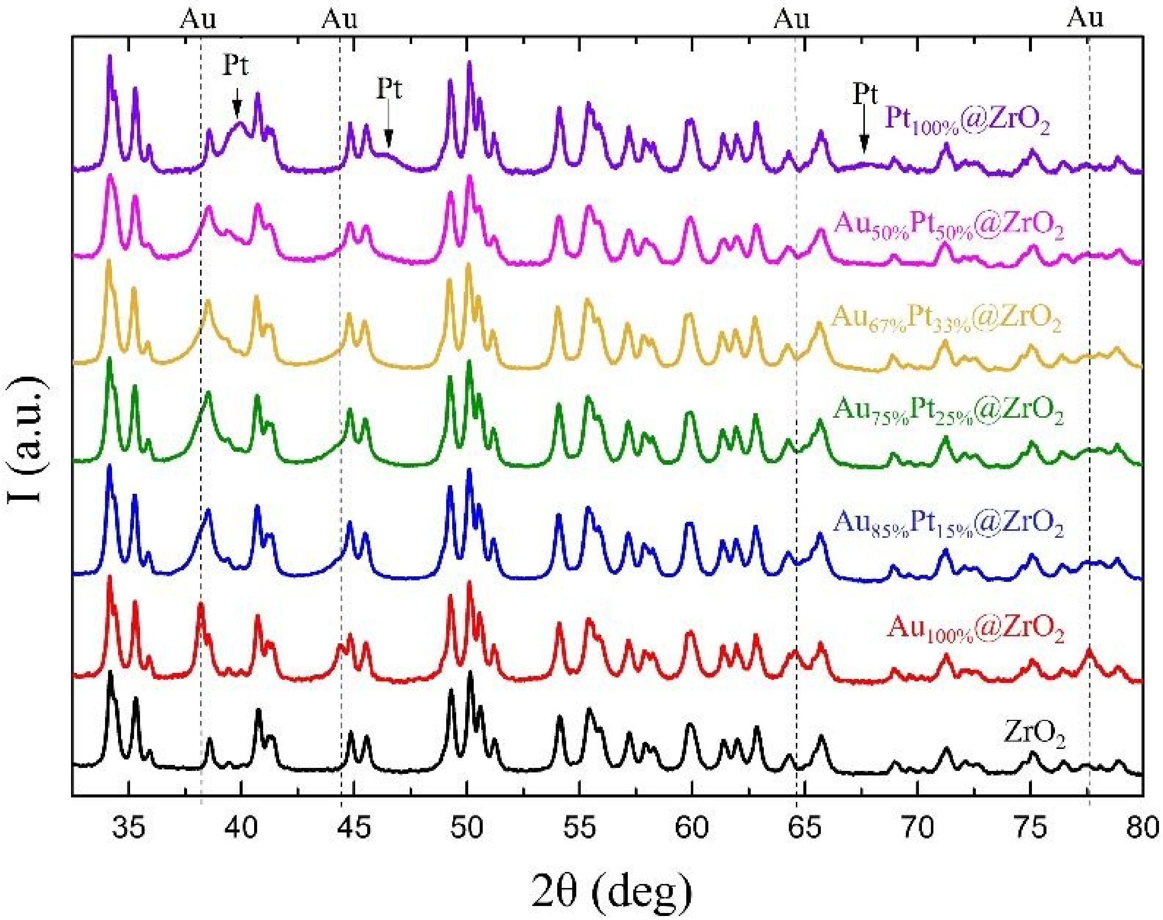

Figure S2. XRD diffractograms of mono- and bimetallic catalysts. The patterns are vertically shifted for clarity. Au reflections are indicated by dashed vertical lines and those of Pt by arrows.

Chemical analysis of the samples.

Table S3. Ratio of elements in the bimetallic samples based on the SEM-EDX spectra using 1.6 (L) and 9.9 (L) spectral lines for gold (Au) and platinum (Pt), respectively.

Sample	Elements	Weight (%)
$Au_{85}\%Pt_{15}\%@ZrO_2$	Au	85
	Pt	15
$Au_{75}\%Pt_{25}\%@ZrO_2$	Au	80
	Pt	20
$Au_{67}\%Pt_{33}\%@ZrO_2$	Au	57
	Pt	43
$Au_{50}\%Pt_{50}\%@ZrO_2$	Au	53
	Pt	47

Microscopic characterization of the samples confirming a homogeneous distribution of deposited particles.

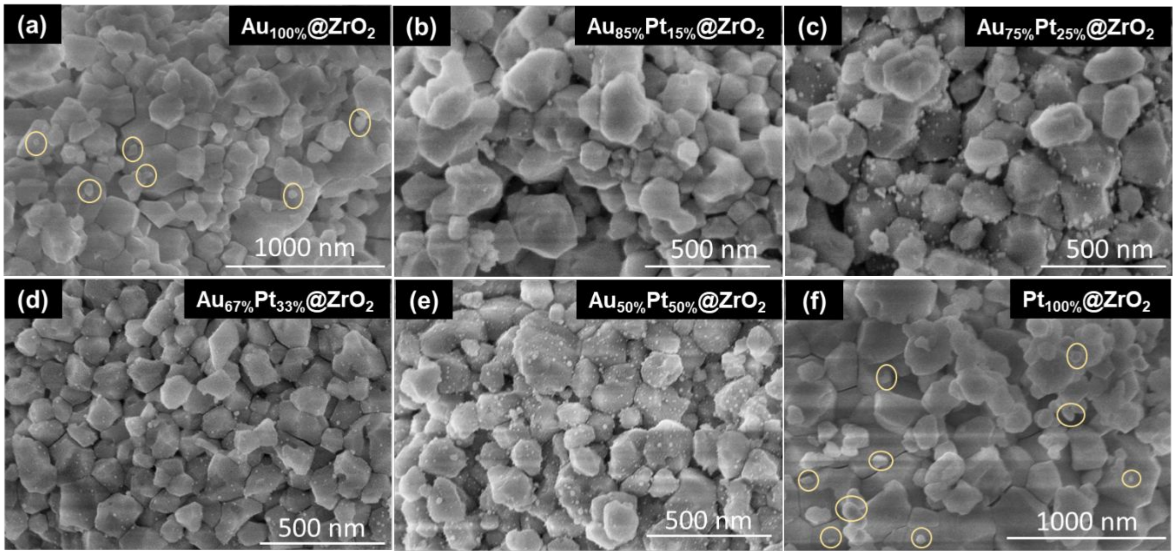

Figure S3. SEM micrographs of the samples (a) $Au_{100\%}@ZrO_2$, (b) $Au_{85\%}Pt_{15\%}@ZrO_2$, (c) $Au_{75\%}Pt_{25\%}@ZrO_2$, (d) $Au_{67\%}Pt_{33\%}@ZrO_2$, (e) $Au_{50\%}Pt_{50\%}@ZrO_2$ and (f) $Pt_{100\%}@ZrO_2$.

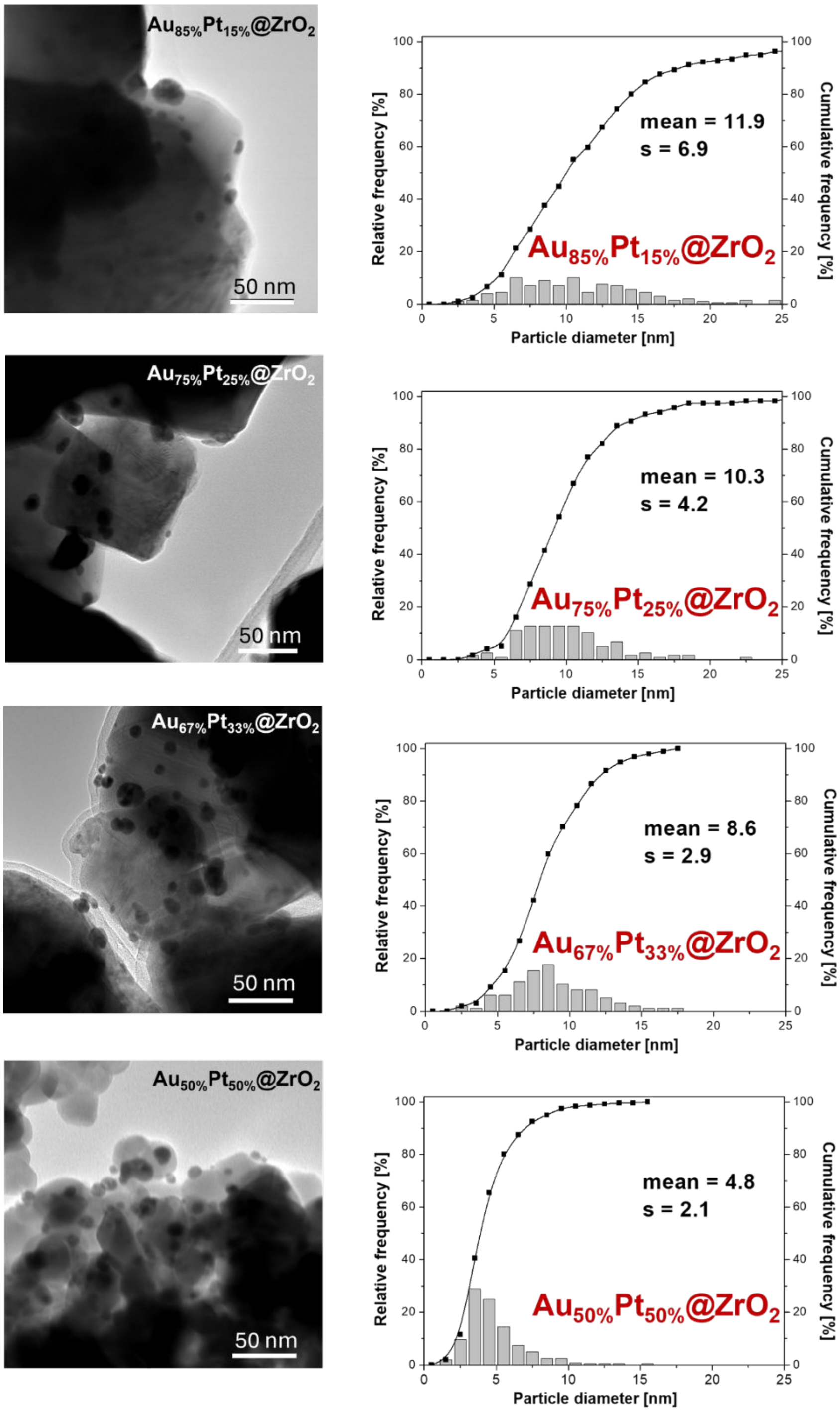

Figure S4. Size distribution of the particles in bimetallic samples.

Surface porosity characterization of the samples.

Table S5. Surface area and pore volume diameter of mono- and bimetallic catalysts based on N_2 adsorption curves.

Catalysts	Surface area S_{BET} (m^2 /g)	Pore diameter (nm)
$Au_{100\%}$ @ZrO_2	4.59	6.32
$Au_{85\%}Pt_{15\%}$@ZrO_2	26.48	9.02
$Au_{75\%}Pt_{25\%}$@ZrO_2	26.79	9.55
$Au_{67\%}Pt_{33\%}$@ZrO_2	27.05	9.45
$Au_{50\%}Pt_{50\%}$@ZrO_2	27.35	8.65
$Pt_{100\%}$@ZrO_2	5.96	6.90
ZrO_2	34.00	-

XPS analysis of the samples.

XPS was performed to confirm the chemical state of the synthesized mono- and bimetallic catalyst. The metal atomic ratios of Au and Pt estimated by XPS are presented in **Table S6**, which were close to the expected ratios. The Au 4f spectra can be fitted with one doublet assigned as Au^0, which were at the BE of the $4f_{7/2}$ and $4f_{5/2}$ peak at around 83.75 and 87.5 eV, respectively. No Au^{3+} peak was observed, which indicates that all the Au precursors are reduced and form metallic nanoparticles. The mono-metallic Pt sample with a higher Pt amount exhibited a broad peak with a peak shape indicating rather the typical asymmetry characteristic for pure bulk Pt. Pt 4f Spectra were successfully fitted with the Pt component, which is separated by the components of Pt $4_{7/2}$ and $4f_{5/2}$ peaks at around 71.22 and 74.52 eV, respectively.

Table S6. Au and Pt (atomic %) calculated based on XPS analysis.

Catalysts	Atomic %	
	Au	Pt
$Au_{100\%}$ @ZrO_2	100	-
$Au_{85\%}Pt_{15\%}$@ZrO_2	67.3	32.7
$Au_{75\%}Pt_{25\%}$@ZrO_2	64.1	35.9
$Au_{67\%}Pt_{33\%}$@ZrO_2	75.8	24.2
$Au_{50\%}Pt_{50\%}$@ZrO_2	66.2	33.8
$Pt_{100\%}$@ZrO_2	-	100

Supporting Information References

[1] Jin, X., et al., *Exceptional performance of bimetallic Pt1Cu3/TiO2 nanocatalysts for oxidation of gluconic acid and glucose with O2 to glucaric acid.* Journal of Catalysis, 2015. **330**: p. 323-329.

[2] Jin, X., et al., *Synergistic Effects of Bimetallic PtPd/TiO2 Nanocatalysts in Oxidation of Glucose to Glucaric Acid: Structure Dependent Activity and Selectivity.* Industrial & Engineering Chemistry Research, 2016. **55**(11): p. 2932-2945.

[3] Lee, J., B. Saha, and D.G. Vlachos, *Pt catalysts for efficient aerobic oxidation of glucose to glucaric acid in water.* Green Chemistry, 2016. **18**(13): p. 3815-3822.

[4] Solmi, S., et al., *Oxidation of d-Glucose to Glucaric Acid Using Au/C Catalysts.* ChemCatChem, 2017. **9**(14): p. 2797-2806.

[5] Derrien, E., et al., *Aerobic Oxidation of Glucose to Glucaric Acid under Alkaline-Free Conditions: Au-Based Bimetallic Catalysts and the Effect of Residues in a Hemicellulose Hydrolysate.* Industrial & Engineering Chemistry Research, 2017. **56**(45): p. 13175-13189.

[6] Monti, E., et al., *Influence of stabilisers on the catalytic activity of supported Au colloidal nanoparticles for the liquid phase oxidation of glucose to glucaric acid: understanding the catalyst performance from NMR relaxation and computational studies.* Green Chemistry, 2023. **25**(7): p. 2640-2652.

[7] Deng, W., et al., *Efficient Catalysts for the Green Synthesis of Adipic Acid from Biomass.* Angewandte Chemie International Edition, 2021. **60**(9): p. 4712-4719.

[8] Khawaji, M., et al., *Composition dependent selectivity of bimetallic Au-Pd NPs immobilised on titanate nanotubes in catalytic oxidation of glucose.* Applied Catalysis B: Environmental, 2019. **256**: p. 117799.

[9] Shi, H., et al., *Oxidation of Glucose Using Mono- and Bimetallic Catalysts under Base-Free Conditions.* Organic Process Research & Development, 2018. **22**(12): p. 1653-1662.

[10] Potrzebowska, N., et al., *Ultrasonication-Assisted Preparation of Au-Pt/ZrO2 Catalysts for the Selective Base-Free Oxidation of Glucose to Glucaric Acid.* ChemCatChem. **n/a**(n/a): p. e202400338.
